# Metagenomic Functional Potential Predicts Degradation Rates of a Model Organophosphorus Xenobiotic in Pesticide Contaminated Soils

**DOI:** 10.3389/fmicb.2018.00147

**Published:** 2018-02-20

**Authors:** Thomas C. Jeffries, Smriti Rayu, Uffe N. Nielsen, Kaitao Lai, Ali Ijaz, Loic Nazaries, Brajesh K. Singh

**Affiliations:** ^1^School of Science and Health, Western Sydney University, Penrith, NSW, Australia; ^2^Hawkesbury Institute for the Environment, Western Sydney University, Penrith, NSW, Australia; ^3^Health and Biosecurity, Commonwealth Scientific and Industrial Research Organisation, North Ryde, NSW, Australia; ^4^Global Centre for Land Based Innovation, Western Sydney University, Penrith, NSW, Australia

**Keywords:** metagenomics, bioremediation, pesticides, soil microbiology, biodegradation, environmental

## Abstract

Chemical contamination of natural and agricultural habitats is an increasing global problem and a major threat to sustainability and human health. Organophosphorus (OP) compounds are one major class of contaminant and can undergo microbial degradation, however, no studies have applied system-wide ecogenomic tools to investigate OP degradation or use metagenomics to understand the underlying mechanisms of biodegradation *in situ* and predict degradation potential. Thus, there is a lack of knowledge regarding the functional genes and genomic potential underpinning degradation and community responses to contamination. Here we address this knowledge gap by performing shotgun sequencing of community DNA from agricultural soils with a history of pesticide usage and profiling shifts in functional genes and microbial taxa abundance. Our results showed two distinct groups of soils defined by differing functional and taxonomic profiles. Degradation assays suggested that these groups corresponded to the organophosphorus degradation potential of soils, with the fastest degrading community being defined by increases in transport and nutrient cycling pathways and enzymes potentially involved in phosphorus metabolism. This was against a backdrop of taxonomic community shifts potentially related to contamination adaptation and reflecting the legacy of exposure. Overall our results highlight the value of using holistic system-wide metagenomic approaches as a tool to predict microbial degradation in the context of the ecology of contaminated habitats.

## Introduction

Environmental contamination by toxic compounds has emerged as a major threat to environmental and human health globally (Singh and Naidu, 2012). With chemical production increasing dramatically each year (Vitousek et al., 1997), much of which is toxic, this threat is likely to worsen unless action is taken to remediate the several million contaminated sites occurring globally, less than 1% of which are currently remediated. Indeed, large-scale chemical contamination has been identified as a “planetary boundary” alongside climate change, ocean acidification, eutrophication, species loss and shifts in nutrient cycling (Rockström et al., 2009).

Efforts to address this problem, and remediate contaminated sites using the metabolic activities of microbes and plants to degrade contaminants *in situ* (Bioremediation), have been hampered by the lack of a holistic system-wide understanding of the complex interactions between degrading organisms and genes, the wider metabolic network of the microbial community, and the environmental variability in each specific habitat (de Lorenzo, 2008). Microbial biodegradation is a complex process, which interacts with nutrient cycling and stress response metabolisms and is dependent on the microbial diversity within each habitat (de Lorenzo, 2008). Thus, there is a need to understand the relationship between microbial community composition and degradation potential and to elucidate which chemical variables and microbial diversity metrics can predict chemical degradation.

As microbes are the primary pollutant degraders in contaminated habitats, understanding microbial processes in individual sites is essential for predicting the best strategy for bioremediation. Degradation may occur via three strategies: natural attenuation, where the community has the metabolic ability to degrade contaminants *in situ* without intervention; biostimulation, where the capability for biodegradation is present but the relevant organisms are at a low abundance or activity and stimulation via amendments such as nutrients or oxygen are required; and Bioaugmentation, where specific cultured microorganisms with the ability to degrade compounds need to be added to the system to ensure degradation (Boopathy, 2000). Currently there is no tool available to aid practitioners in making the decision of which is the optimal strategy for remediation, thus the development of a predictive approach informed by an understanding of microbial composition and metabolic potential is a key goal of bioremediation. Only recently have the ecogenomic tools emerged to allow for this information to be profiled directly in the environment via next-generation DNA sequencing supported by tools such as network analysis which allow the interactions between microbial taxonomy, function and environmental variables to be visualized at the metabolic level (Fuhrman and Steele, 2008; Hugenholtz and Tyson, 2008; Fuhrman, 2009).

Organophosphorus pesticides (OP) are among the most widely used classes of chemicals in the agriculture and chemical manufacturing industries (Singh and Walker, 2006; Whitacre, 2012). Globally 4.6 million tons of chemical pesticides are annually sprayed into the environment (Zhang et al., 2011), 38% of which are organophosphorus compounds (Singh and Walker, 2006). With the world's population expected to grow from 6.8 billion today to 9.1 billion by 2050 with limited croplands (Alexandratos and Bruinsma, 2012), further intensification of the use of pesticide to increase crop production in order to ensure food security is likely (Tilman et al., 2001; Zhang et al., 2007, 2008). Despite there being negative effects of pesticide use (described below), they are currently essential for sustaining agriculture and are a critical factor in global food security, particularly in developing countries (Carvalho, 2006). The success of these compounds is a result of their high toxicity for insects and target organisms however they also can poison non-target organisms. OP pesticides have high mammalian toxicity and are responsible for several million poisonings and 300,000 deaths annually (Singh, 2009), which are often a result of both accidental and intentional release of agricultural pesticides. Organophosphorus compounds are also common chemical weapon agents, of which ~200,000 tones remain stored (Singh, 2009) and which potentially will require disposal and decontamination under the Chemical Weapons Convention 1993, in addition to the vast amount of agricultural stockpiles and contaminated storage vessels that require eventual remediation.

As they are prone to rapid degradation in some environments (Singh and Walker, 2006), understanding the factors that facilitate their short term effective use, but prevent long-term contamination, is an essential element of sustaining agriculture. Organophosphorus compounds are considered highly biodegradable, and several phylogenetically distinct taxa have been isolated that are capable of degrading them via a series of enzymatic pathways mediated by phosphoesterase enzymes (Singh, 2009); however, how this process takes place *in situ* and in the context of the overall metabolic network of environmental samples is unknown. Understanding how this occurs in specific soils and predicting which sites are particularly prone to microbial degradation can save millions of dollars for both farmers and pesticide companies, and can ensure the correct timeframe for use if applied in a sustainable fashion.

To address these knowledge gaps, and to integrate ecogenomic tools and degradation studies in the field to predict biodegradation efficiency and support the remediation strategy decision making process, we have used metagenome sequencing to profile the functional potential and taxonomic community composition of soils with a history of OP pesticide exposure. We hypothesized that a system-wide profile of microbial metabolism can be linked to OP pesticide degradation rates in soils despite differing exposure histories.

## Materials and methods

### Site selection and soil sampling

Soil was collected from five sugarcane farms in Queensland Australia. Three of the sites (sites 1, 2, and 4) were from the Burdekin region, Site 3 was from the Mackay region and Site 5 was from the Tully region (Supplementary Table 3, Rayu et al., 2017). All sites have some history of agricultural pesticide usage with the application of the organophosphate chlorpyrifos (CP). Sites from Burdekin and Tully (1, 2, 4, 5) had been exposed to CP annually; however, 13 years ago developed a loss of pest-control efficacy in controlling target pests potentially due to high field rates of degradation necessitating the shift to a non-organophosphorus pesticide. The Mackay sampling site (3) is still exposed occasionally to CP as an effective tool to control pests. At each site sampling was undertaken within two plots: one several rows within the crops that had been exposed to pesticide directly (termed R in our analysis) and one that was located several meters from the crop and that had received indirect contamination from runoff and wind (termed H in our analysis). These two plots were chosen to encompass a gradient of pesticide input to better capture the variety of states in which pesticide persists in the environment. Triplicate samples were collected at each subplot (H or R) and each of these replicates consisted of three pooled random cores. Fresh soil was sieved through 2 mm to separate vegetation and coarse particles from the sample at stored at either −20°C for DNA extraction or 4°C for degradation assays. Soils directly from the field were used for shotgun sequencing as described below.

### Soil DNA extraction and shotgun sequencing

DNA was extracted from 10 g homogenized soil using bead beating and chemical lysis (PowerMax® soil DNA Isolation Kit Mobio, USA) following the manufacturer's protocol. Genomic DNA concentration was quantified using a Qubit 2.0 fluorometer (Invitrogen). A shotgun metagenomic library was generated and sequenced using Illumina® HiSeqTM at the Hawkesbury Institute for Environment NGS research center utilizing TruSeq library preparation.

### Metagenome processing, annotation, and statistical analysis

**Figure 1:**
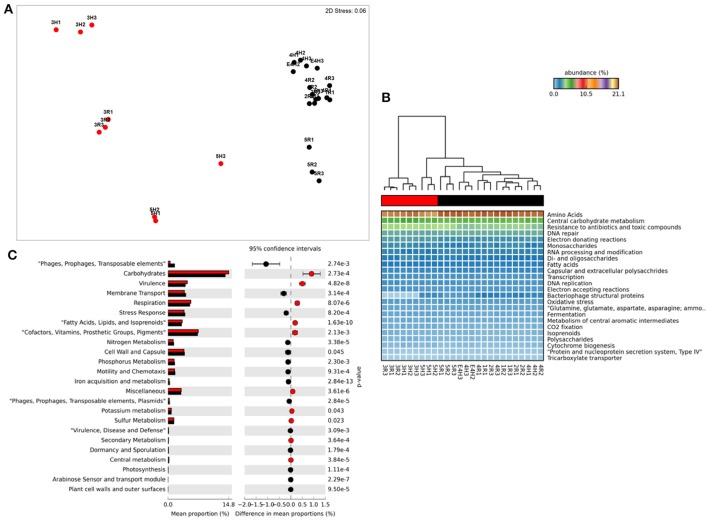
Functional metagenome profiles: **(A)** non-Metric Multidimensional Scaling (MDS) plot of metagenome functional profile similarity (Bray-Curtis), **(B)** Heatmap of functional pathway abundance with samples grouped by similarity (UMPGA clustering) and pathways ranked by mean abundance, **(C)** extended error bar plot of pathways differentially abundant between sample clusters (>0.05% difference in abundance, *p* < 0.05, Welch's *t*-test). Color of circles and dendrogram bar denotes sample grouping (Red, slow degrading; Black, rapid degrading).

**Figure 2:**
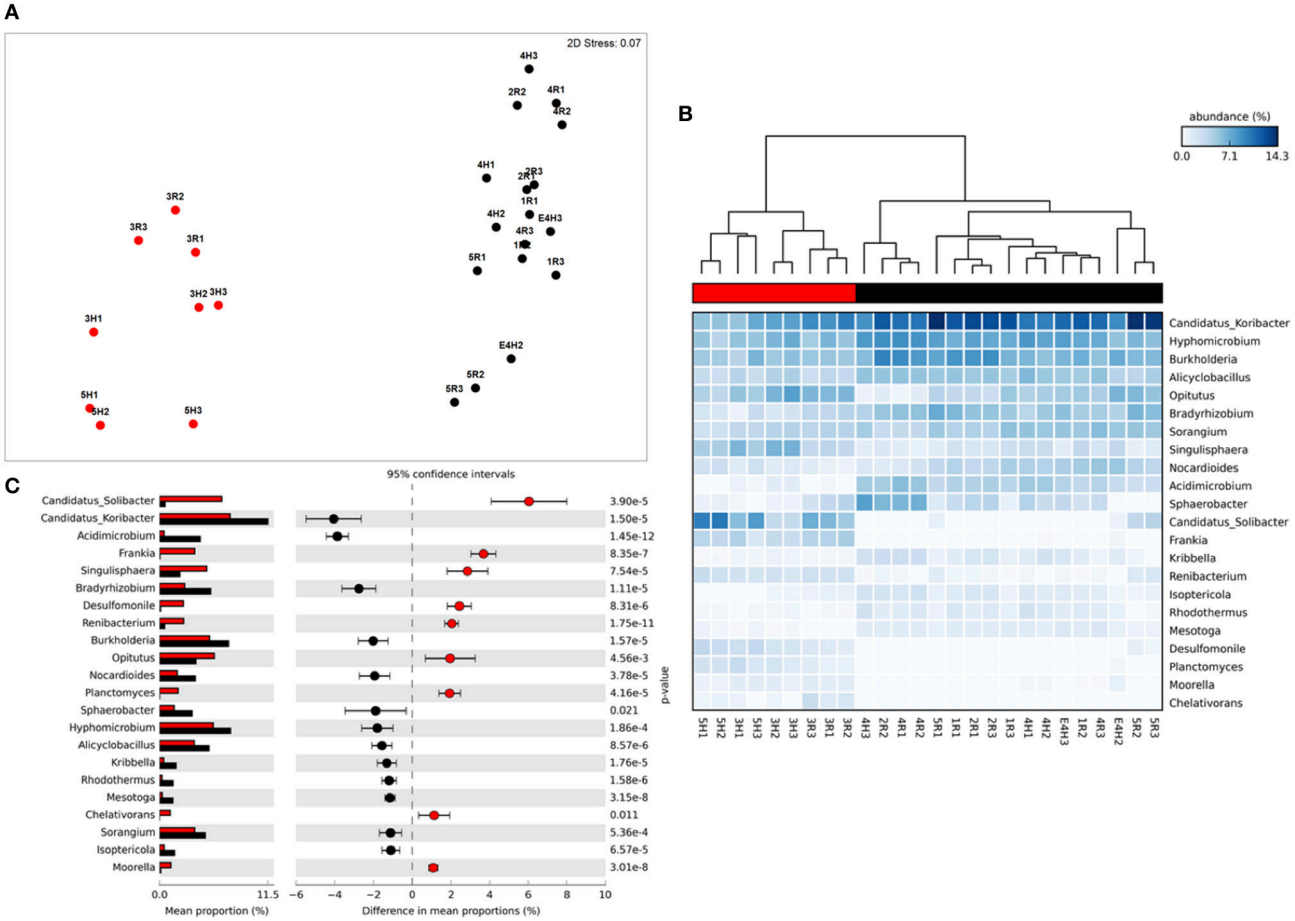
Taxonomic metagenome profiles: **(A)** non-Metric Multidimensional Scaling (MDS) plot of taxonomic profile similarity (Bray-Curtis), **(B)** Heatmap of genera abundance with samples grouped by similarity (UMPGA clustering) and taxa ranked by mean abundance, **(C)** extended error bar plot of genera differentially abundant between sample clusters (>1% difference in abundance, *p* < 0.05, Welch's *t*-test). Color of circles and dendrogram bar denotes sample grouping (Red, slow degrading; Black, rapid degrading).

**Figure 3:**
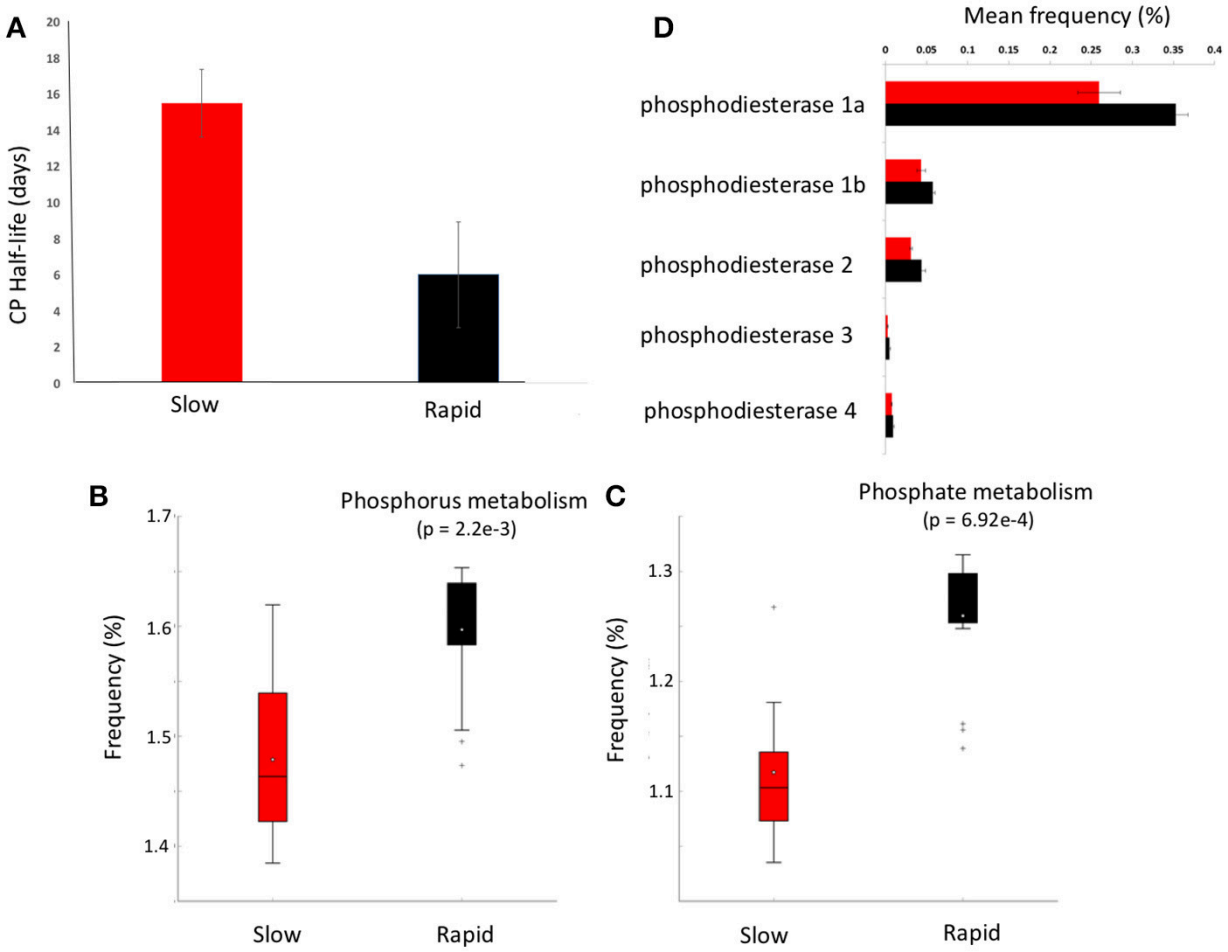
Chlorpyrifos (CP) degradation potential. **(A)** Mean half-life of CP after the third application of pesticide to each soil in samples forming clusters in metagenomic ordinations, boxplots of metabolic function abundance for **(B)** phosphorus metabolism, **(C)** phosphate metabolism (*p* < 0.05), and **(D)** relative abundance of phosphodiesterase enzymes in clusters (*t*-test of differences in abundance *p* < 0.05). Error bars = SD and red represents slow degrading cluster, black represents rapid degrading cluster. Phosphodiesterase 1a and 1b, diguanylate_cyclase/phosphodiesterase_(GGDEF_&_EAL_domains_with_PAS/PAC_sensor(s); 2, Glycerophosphotyl_diester_phosphodiesterase_[EC_3.1.4.46); 3, 2',3'-cyclic-nucleotide_2'_phosphodiesterase_[ec_3.14.16]; 4, Alkaline_phosphodiesterase_1_[EC_3.1.4.1__Nucleotide_pyrophosphatase_(ec_3.6.1.9).

**Figure 4:**
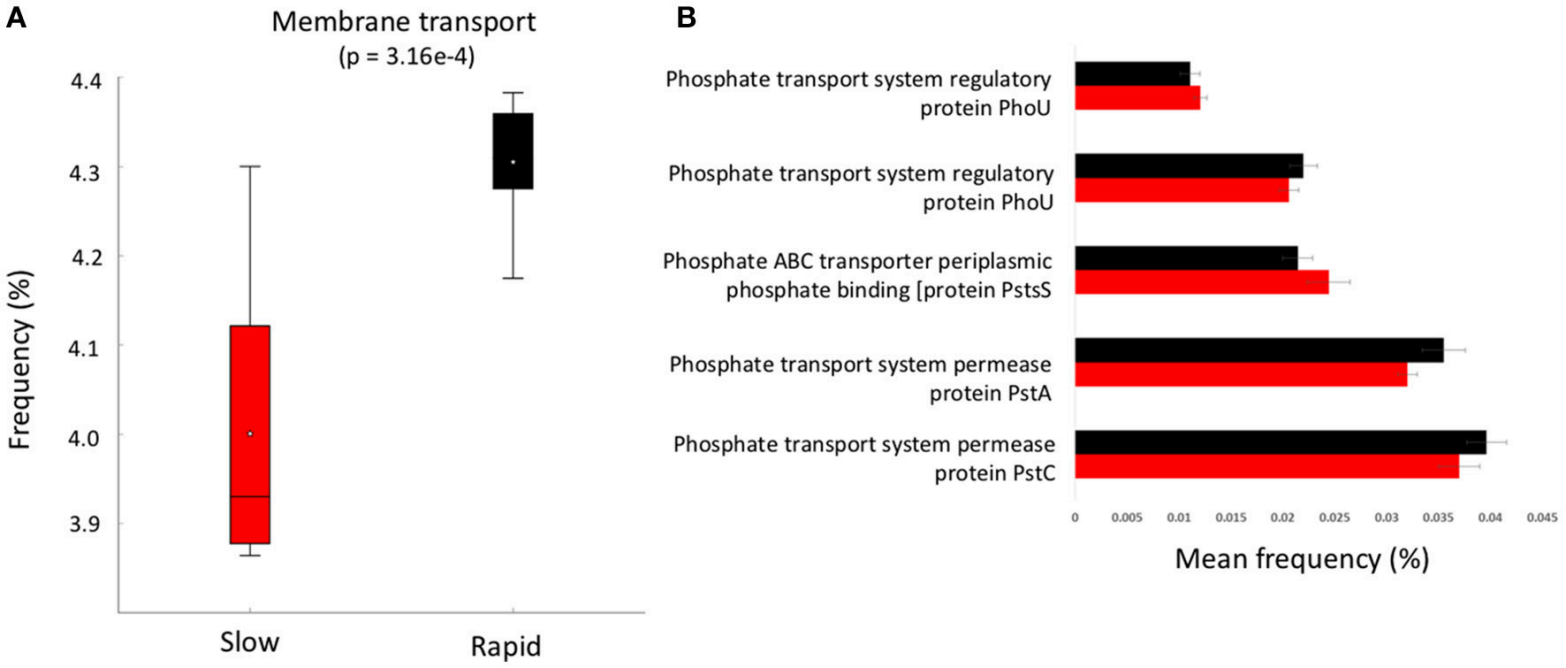
**(A)** boxplot of metabolic function abundance for membrane transport and **(B)** abundance of phosphorus transport pathways (>0.01% abundance, *t*-test of differences in abundance *p* < 0.05). Error bars = SD and red represents slow degrading cluster, black represents rapid degrading cluster.

Reads were adapter filtered and quality trimmed to remove regions with a quality score of <Q25 using SeqPrep (https://github.com/jstjohn/SeqPrep). Following QC we used 564,033,668 forward reads for analysis with an average read length 0 f 150 bp. This totaled ~84 GBp of data, with an average of 3.5 GBp per sample. These unassembled reads were annotated using the FOCUS pipeline (Silva et al., 2014) to determine taxonomy and the SUPER-FOCUS pipeline (Silva et al., 2016) to determine metabolic potential in both cases using the SEED database as a reference (Overbeek et al., 2014). Both of these tools utilize K-mer frequencies and non-negative least squares to optimize database query efficiency. SUPER-FOCUS utilized the RAPSearch2 algorithm (Zhao et al., 2012) for database comparison with outputs being normalized by sequencing effort. Taxonomic and metabolic profiles consisting of relative abundances at each hierarchical level of the SEED database were imported into the PRIMER software package (Clarke, 1993; Clarke and Gorley, 2006) and square root transformed. The Bray-Curtis similarity between profiles was ordinated using non-metric MultiDimensional Scaling (MDS) with the significance of groupings assessed using ANOSIM with 999 random permutations (visualized in Figures 1A, 2A). Additional statistical analysis and visualization was conducted using the STatitstical Aanalysis of Metagenomes (STAMP) package (Parks et al., 2014) using the heatmap function with UMPGA clustering to produce dendrograms. In these plots (Figures 1B, 2B) the relative color specifies the abundance of individual categories with the dendrogram displaying the beta-diversity patterns between samples based on these variables. The significance of abundance differences between clusters was determined using Welch's *t*-test, which is optimized version of the Student's *t*-test for samples with unequal variance, with the output of these tests visualized as extended error bar plots (CI = Welch's inverted 95%) or box-plots within STAMP which display the *t*-test result and variance of the data (Figures 3B,C, 4A). In the extended error bar plots the relative abundance of each category is specified for both sample groupings as bars, with the difference in proportions with 95% confidence interval error bars, are displayed for each category on the right of the plot (Figures 1C, 2C). Additionally specific pathways potentially relating to phosphorus metabolism and membrane transport were directly visualized as bar charts (Figures 3D, 4B) with the relative abundance of pathways and standard deviation values being extracted from the STAMP output statistics table.

**Figure 5:**
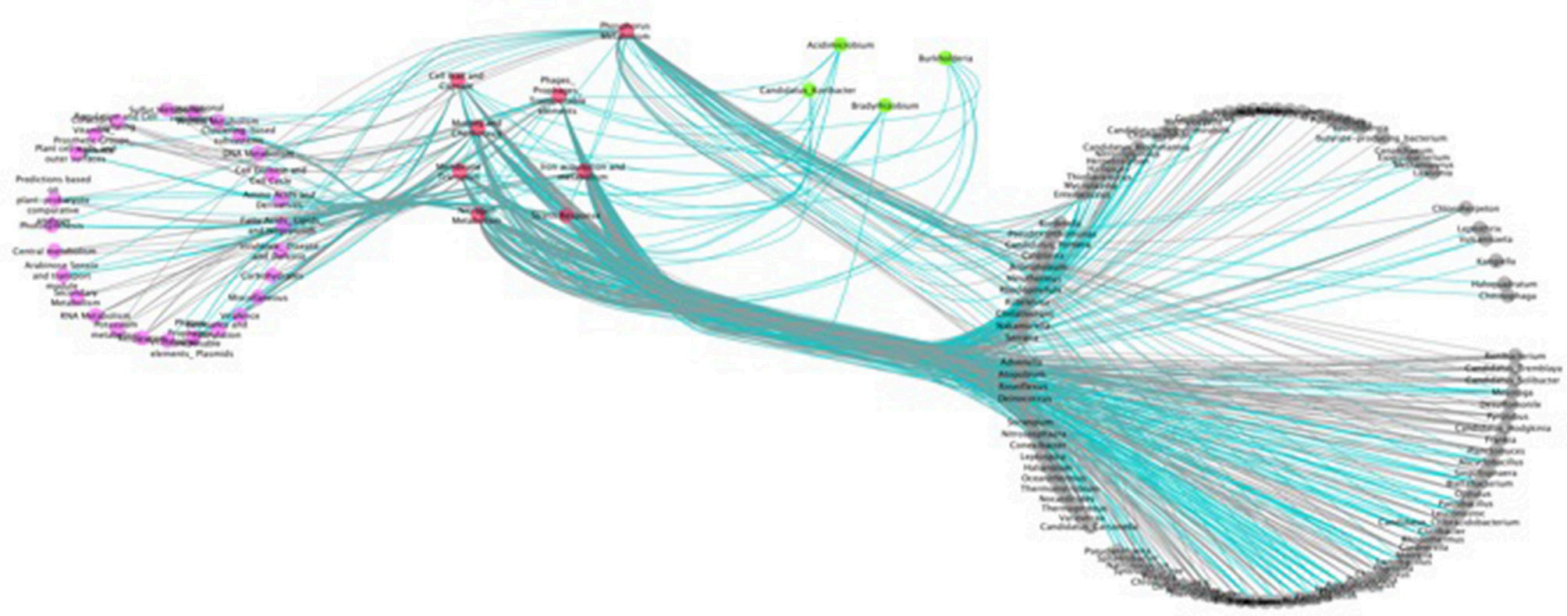
Network analysis of variable interactions with top drivers of taxonomic and functional dissimilarity. All edges are statistically significant (*p* < 0.05) based on Maximal Information Coefficient (MIC) score. Pink nodes, metabolic category; dark pink nodes, metabolic categories found to be top drivers of clustering *(t*-test); gray nodes, taxa (genus); green circles, genera found to be top drivers of clustering (*t*-test). Black edges, positive interactions; blue, negative interactions.

To explore the system-wide interactions between variables we applied network analysis. Interactions were determined using the Maximal Information-based Non-paramteric Exploration (MINE) algorithm (Reshef et al., 2011). MINE calculates the strength of the relationship between each individual variable (MIC score) in addition to descriptors of the relationship such as linearity and regression. Only variables with values for >50% of samples were included and the dataset was filtered to include only significant (*p* < 0.05) correlations. All samples in the dataset were included in the analysis. Results were visualized with Cytoscape V3 (Shannon et al., 2003) with variable interactions displayed in Figure 5.

### Alpha-diversity

The alpha-diversity (Shannon Index) of metagenomic profiles was calculated on biom formatted output tables from FOCUS/SUPERFOCUS using the QIIME software package (Caporaso et al., 2010).

### Microbial abundance

Overall bacterial abundance was determined by amplifying 16S rRNA gene on a rotor-type thermocycler (Corbett Research; Splex) using primer pair Eub338F (5'ACTCCTACGGGAGGCAGCAG 3') and Eub518R (5'ATTACCGCGGCTGCTGG 3') (Fierer et al., 2005). This primer set targets and amplifies the 16S rRNA gene present in all the soil bacterial groups. Reactions were performed in 20 μl volumes using™ SYBR® No-ROX Kit (Bioline Reagents Ltd.) as described with changes in PCR conditions. After initial denaturation at 95°C for 3 min, PCR conditions were as follows: 40 cycles of 10 s at 95°C, 20 s at 53°C and 20 s at 72°C. An additional 15 s reading step at 83°C was added at the end of each cycle.

### Organophosphorus degradation assays

To determine the organophosphorus pesticide degradation potential of soils, a commercial formulation of chlorpyrifos 500 EC (500 g/L, Nufarm) was applied to 250 g of soil from all sites in plastic jars and mixed to a final concentration of 10 mg/kg (Rayu et al., 2017). The water holding capacity of the soil was adjusted to 40% and was maintained by regular addition of Milli Q water. Each treatment was performed in triplicate. The screw cap plastic jars containing the treated soil were incubated and maintained under aerobic conditions, in the dark at room temperature. All the soil-pesticide combinations were sampled periodically up to 105 days to determine the microbial properties and degradation of pesticides. After 45 days, or when more than 75% of the initial concentration of the pesticides disappeared, another spike of CP was applied to the soil at final concentration of 10 mg/kg. The soils were retreated with the third application of pesticide (10 mg/kg) 50 days after the second treatment, when maximum degradation of pesticide took place. These second and third additions of CP were added to assess if the soil microbial community was still able to degrade the compound following repeated exposures and if the degradation rate would increase as a result of metabolic adaptation. Further details of this degradation kinetics experiment are provided in Rayu et al. (2017). In addition to this, soil samples (25 g) treated with antibacterial and antifungal agents, chloramphenicol and cycloheximide (1 ml each; 5 mg/ml in water), respectively (Singh et al., 2003) were also maintained as controls.

Following incubation, chlorpyrifos and it's metabolites were extracted from soil (2.5 g) by mixing with acetonitrile:water (90:10, 5 ml) in McCartney glass vials. The vials were vortexed and pesticide extraction was conducted by shaking the mix for 1 h on a shaker (130 rpm). The samples were centrifuged for 5 min at 15,000 rpm and the supernatant was filter sterilized through a 0.22 μm nylon syringe filter for High Performance Liquid Chromatography (HPLC) analysis using an Agilent 1,260 Infinity HPLC system. CP and IC were separated on Agilent Poroshell 120 column (4.6 × 50 mm, 2.7 μm) with Agilent ZORBAX Eclipse Plus-C18 guard column (4.6 × 12.5 mm, 5 μm) (Singh et al., 2003). The injection volume was 10 μl and the mobile phase was acetonitrile:water (75:25), acidified with 1% phosphoric acid. The analytes were eluted at 40°C with isocratic mobile phase flow rate of 0.8 ml/min for 4.5 min. The pesticides were detected spectrophotometrically at 230 nm. Pesticide degradation was ascribed by the first-order function (Ct = Co × e-kt). The half-lives of the pesticides were obtained by function t1/2 = ln2/k. Each value is a mean of three technical replicates (*n* = 3). The half-lives were displayed as bar plots (Figure 3A).

## Results

Overall we profiled the metagenomes of two sub-plots from five sugarcane farms with differing histories of pesticide application. To determine the influence of the different legacy of pesticide exposure on contemporary microbial composition and to predict degradation potential, we profiled the metagenomic potential of the soils from these sites.

### Functional analysis of metagenomic profiles

Ordination of functional profiles based on the relative abundance of metabolic pathways (SEED level 2, Figure 1A) demonstrated that samples formed two distinct clusters; one consisting of soils from the two Mackay fields (3H and 3R) and a field from Tully (5H), and a second cluster consisting of the remaining samples. As the cluster containing soils from 3H, and 3R was largely composed of soils with historically effective control of pests in the field (with the exception of 5H), this cluster was termed “slow degradation” with the other cluster (sites 1H, 1R, 2H, 2R, 4H, 4R, 5R) termed “rapid degradation,” which has had no exposure to OP pesticides for several decades due to a loss of pest-control efficiency. These clusters were strongly supported by ANoSIM analysis (Global *R* = 0.94, Sig. = 0.1%) indicating that the grouping was highly significant. With the exception of the samples from Mackay (Site 3), no evidence of a geographic or spatial pattern in ordination was evident.

This clustering was consistent with the sample grouping dendrogram of high level metabolic pathway abundance (SEED level 1, Figure 1B), which showed that all samples were dominated by core housekeeping genes such as amino acid and carbohydrate metabolism. Resistance to antibiotics and toxic compounds was also abundant and showed differences in magnitude between samples and clusters, as did many less abundant metabolisms (Figure 1B). To further identify which metabolic pathways were differentially abundant between sample clusters we conducted Welsh's *t*-test to compare the mean abundance of metabolic pathways (Figure 1C). The slow degradation cluster had significantly higher abundances of virulence genes as well as housekeeping pathways such as carbohydrate and fatty acid metabolism and respiration. The rapid degradation cluster had more abundant genes belonging to phage and transposable elements as well pathways for membrane transport, stress response, motility and chemotaxis and key nutrient cycles such as phosphorus, nitrogen and iron metabolism.

At level 2 of the SEED database hierarchy, 116 out of 194 metabolic pathways were significantly (*p* < 0.05) overrepresented in one of the two clusters (Supplementary Table 1). This equates to 60% of the metabolic pathways indicating a wholesale shift in microbial metabolic potential between these two groups of soils. To investigate how these contributed to the dissimilarity between clusters (Figure 1A) we conducted SIMPER analysis (Clarke, 1993). For metabolic pathways, the top 10 drivers were responsible for 35% of the overall dissimilarity between samples (Table 1). Similarly to the *t*-tests conducted at higher level metabolic groupings (SEED Level 1), these pathways came from a variety of core and adaptive metabolisms with the slow degradation cluster being defined by an increase in genes for sugar acquisition, carbon fixation and resistance to antibiotics and toxic compounds. The rapid degradation cluster had an increase in genes involved in transport and phage.

**Table 1:** SIMilarity PERcentage (SIMPER) analysis of the 10 most significant drivers of clustering between soil clusters.

	**Abundance group one (%)**	**Abundance group two (%)**	**Contribution to dissimilarity (%)**
**FUNCTIONAL PATHWAY (SEED LEVEL 2)**			
Bacteriophage structural proteins	0.18	1.56	17.57
Tricarboxylate transporter	0.06	0.15	3.07
Resistance to antibiotics and toxic compounds	4.08	3.50	2.97
Di- and oligosaccharides	2.16	1.88	2.02
Protein and nucleoprotein secretion system, Type IV	0.15	0.23	1.78
Electron donating reactions	2.66	2.43	1.52
Cytochrome biogenesis	0.52	0.41	1.51
Uni- Sym- and Antiporters	0.06	0.10	1.50
Phages, Prophages	0.12	0.17	1.46
CO_2_ fixation	0.77	0.66	1.39
**GENUS**			
Candid atus_Solibacter	6.40	0.12	3.65
Frankia	3.69	0.01	3.06
Acidimicrobium	0.23	4.28	2.65
Desulfomonile	2.50	0.01	2.45
Planctomyces	1.90	0.00	2.24
Sphaerobacter	1.19	2.66	1.67
Renibacterium	2.56	0.36	1.67
Syntrophobacter	0.92	0.01	1.55
Moorella	1.19	0.03	1.52
Chelativorans	0.81	0.00	1.50

*Features higher in the “slow degradation cluster” in red font, features higher in “rapid degradation cluster” in black font*.

### Taxonomic analysis of metagenomic profiles

The sample grouping observed for functional profiles (Figure 1) was also strongly reflected in the taxonomic profile of soil metagenomes (Figure 2A) that showed an even stronger sample partitioning (ANOSIM Global *R* = 9.97, Sig. = 0.1%) indicating that taxonomic and metabolic profiles were tightly coupled in these soils. As for metabolism, this clustering was consistent with the sample grouping dendrogram of taxa abundance (Figure 2B), which showed that samples were dominated by *Koribacter, Hyphomicrobium*, and *Burkholderia*, particularly those samples in the rapid degradation group, with *Solibacter* showing a high abundance in soils from the slow degradation group. Overall, bacterial genera were variable in abundance between samples and clusters (Figure 2B). To further identify which taxa were differentially abundant between sample clusters we conducted Welsh's *t*-test to compare the mean abundance of genera (Figure 2C). Overall, 86 out of 194 genera were significantly (*p* < 0.05) overrepresented in one of the two clusters (Supplementary Table 2). This equates to 43% of these taxa indicating a strong shift in community composition between two these groups of soils. Of the taxa that differed most in abundance between the clusters (Figure 2C), abundant bacteria such as *Solibacter, Singulisphaera*, and *Desulfomonile* were higher in the slow degrading cluster. In the rapid degradation cluster the abundant *Koribacter* and *Acidomicrobium* as well as *Bradyrhizobium* and *Burkholderia* most differed in abundance compared to the slow degrading cluster. With the exception of *Singulisphaera* and *Bradyrhizobium*, these taxa were among the top drivers of the clustering between samples (Figure 2A) as identified using SIMPER analysis, the top 10 genera of which were responsible for 22% of the overall dissimilarity between groups (Table 1).

### Organophosphorus degradation potential

Given the varying legacy of pesticide exposure and community dissimilarity between soils, we conducted microcosm experiments to determine the contemporary microbial degradation kinetics of a model organophosphorus pesticide, chlorpyrifos, in each soil (Figure 3A). The half-life of chlorpyrifos in soils ranged from 3 to 17 days with the mean half-life of contemporary degradation being significantly higher in the three soils which form the discrete “slow degradation cluster” for both functional and metabolic profiles (3H, 3R, and 5H, Figures 1, 2). Surprisingly, sample 5H, which comes from a site with a reported loss of pesticide efficacy, clustered with 3H and 3R which also showed a slow rate of degradation and on which chloropyrifos is still applied. The soils that formed the “rapid degradation cluster” of the ordinations showed a higher contemporary rate of pesticide degradation.

To further investigate the genes involved in phosphorus metabolism, we mined the metabolic profiles for functional pathways and genes potentially involved in degradation. Metabolic pathways for phosphorus metabolism (SEED level 1) and phosphate metabolism (SEED Level 2) were both significantly higher in samples forming the enhanced degradation cluster, than the slow degradation cluster (*p* < 0.05, Figures 3B,C). Rapid degradation soils also showed a higher abundance of genes encoding phosphodiesterase enzymes (*p* < 0.05, Figure 3D) which cleave phosphodiester bonds present in organophosphorus and play a major role in pesticide degradation (Singh and Walker, 2006) among other functions. Additionally, the potential for membrane transport generally (Figure 4A) and phosphate transport specifically, for the majority of the most abundant transport genes (Figure 4B) is higher in soils which form the rapid degradation cluster, indicating an overall shift in this community to favor the transport and metabolism of phosphorus compounds within their lifestyle.

Overall, metagenome alpha diversity showed a negative relationship to degradation rates, with more diverse samples having longer half-lives of chloropyrifos, and microbial abundance, assayed using 16S rDNA concentration, showed a positive relationship with degradation rates of samples (Supplementary Figures 1, 2) supporting the view that microbial community composition plays a major role in the dynamics of chloropyrifos in these soils.

### Network analysis

To investigate the interactions and co-occurrence patterns between metabolic and taxonomic variables we conducted a network analysis (Figure 5, Supplementary Figures 2, 3). Overall there was strong connectivity between functional categories and taxonomic groups (Supplementary Figure 2). In particular, variables that were the most elevated in the rapid degrading cluster (Figures 1C, 2C) were strongly associated with each other, and highly interconnected to diverse taxa and metabolisms, showing highly similar co-occurrence patterns (Figure 5).

Overall, soils that were found to have higher degradation rates of CP were found to have similar overall metabolic and taxonomic metagenome profiles different from those soils that retained CP longer. This was a result of abundance shifts in diverse taxa and metabolic categories including those potentially involved in organophosphorus degradation.

## Discussion

Whilst many studies have analyzed individual catabolic genes involved in degradation of contaminants (de Lorenzo, 2008; Ufarté et al., 2015) including pesticides (Li et al., 2008; Singh, 2009; Imfeld and Vuilleumier, 2012), and have applied metagenomics to assess the influence of contamination on functional potential (Hemme et al., 2010; Mason et al., 2012; Smith et al., 2013), this is the first study to use metagenomics to demonstrate that system-wide responses in the context of the degradation potential of the soils demonstrated experimentally. This has provided key insights into the ability of differential microbial profiles to predict the degradation potential of organophosphorus compounds in different soils, within the context of the wider metabolic potential of communities and adaptation to local conditions and contaminants.

### Functional metabolic potential

We observed that soils with a higher degradation potential support a differing metabolic potential to those in which organophosphorus is retained for longer. Overall differences in the relative abundance of high-level metabolic categories suggested that microbes in more rapidly degrading soils had a higher functional capacity in terms of nutrient cycling, with an increased abundance in pathways for nitrogen, phosphorus and iron metabolism coupled to increased transport and stress response genes. Overall this indicated a more adaptive community better able to sustain nutrient cycling and microbial activity, potentially enabling the increased degradation potential of these soils. In particular, the increased ability to metabolize and transport phosphorus and phosphate compounds could enhance the catabolism of OP compounds in these soils and provide direct nutritional benefits to soil microbiota. Hydrolytic cleavage of phosphate ester bonds in OP compounds has been suggested as a nutrient acquisition strategy in many environments (Chen et al., 1990; Singh and Walker, 2006; White and Metcalf, 2007; Hirota et al., 2010) and is supported here by an increased abundance in phosphoesterase enzymes in soils with increased degrading potential.

Phosphodiesterase enzymes are directly involved in some organophosphorus degradation (Singh and Walker, 2006) and have been isolated from diverse degrading organisms (Singh, 2009). Whilst they also play a role in other cellular processes such as nucleic acid and cAMP metabolism, their increased abundance here indicates increased potential for OP metabolism. Other enzymes potentially involved in OP degradation, such as phosphotriesterase were not abundant presumably due to their rarity in the environment generally, meaning they were potentially overlooked at this level of sequencing depth and their relative scarcity in sequence databases. In addition to genes directly involved in OP compound degradation, metabolic pathways potentially involved in enhancing the ability of microbes to access and transport OP compounds were found to display abundance shifts. For example, an increase in genes related to motility and chemotaxis may also play a role in the increased degradation potential in rapid degradation soils as the success of the microbial degradation of pollutants is often limited by the inability of the bacteria to access contaminant molecules (Fernández-Luqueño et al., 2011; Niti et al., 2013) which act as chemoattractants in contaminated habitats (Samanta et al., 2002; Parales, 2004; Kato et al., 2008; Ahemad and Khan, 2011). Therefore, bacterial chemotaxis provides a distinct advantage to the motile bacteria in finding their substrate and degrading them at higher rates (Pandey and Jain, 2002).

Interestingly, the most overrepresented functional category in rapid degradation soils was phage genes, including both structural and lateral gene transfer related pathways. Whilst little work has been conducted regarding the role of viruses in contamination response and degradation, metagenomics studies in other habitats have demonstrated that phage carry diverse accessory genes enabling microbial communities to be more metabolically variable and adaptive to environmental change (Dinsdale et al., 2008). Additionally, phage genes have been found in high abundances during hydrocarbon bioremediation and have been implicated in controlling the microbial loop via lysis (Rosenberg et al., 2010). Overall, network analysis revealed that many of these functions found to be overrepresented in the rapid degradation cluster were highly connected to other diverse metabolisms and genera. This indicates that a shift in the abundance of these variables may have far reaching metabolic consequences throughout the community and *vice versa* that shifts in microbial diversity will influence the ability of the community to degrade contaminants.

The high abundance of core house-keeping genes in the soils with slow degradation of OP is consistent with the high abundance of these genes in the majority of habitats (Dinsdale et al., 2008; Hewson et al., 2009; Smith et al., 2012; Tout et al., 2014) and could indicate a less adaptive community with less abundant specialized metabolic processes. By contrast, a higher abundance of more adaptive genes in group two soils is consistent with a higher genomic flexibility and abundance of specialist metabolic accessory genes documented in other stressed or contaminated environments (Ford, 2000; Paul et al., 2005; Ahmed and Holmström, 2014).

Based on the microbial degradation results, when pesticide was introduced into soils rapid degradation was observed in soils corresponding to one metagenome cluster but not the other even after repeated application. Such predictive knowledge is critical to develop effective decision support for efficient bioremediation and to predict the type of bioremediation strategy which would be most efficient. For example, soils which form the “rapid degradation” cluster could be self-remediated *in situ* (natural attenuation) without intervention; however, those in the “slow degradation” cluster, which had a metabolic profile less well suited to degradation, may benefit form a bio-augmentation or bio-stimulation approach to expedite site remediation.

### Taxonomic community composition

Microbial community responses to pesticide contamination have been studied previously (Baxter and Cummings, 2008; Wang et al., 2008; Floch et al., 2011; Imfeld and Vuilleumier, 2012; Zabaloy et al., 2012) with potential patterns representing microbial adaptation to contamination; however, few studies have employed metagenomics for this purpose (Imfeld and Vuilleumier, 2012). We found several key taxa to be overrepresented in the group two soils able to degrade OP more efficiently indicating a linkage between community composition and pesticide degradation and tolerance. One of the most abundant taxa that were overrepresented in cluster two was the genera candidatus *Koribacter* that is a common versatile heterotrophic soil bacterium first isolated in Australian agricultural soils (Davis et al., 2005). Genomic studies suggest that these can metabolize complex carbon substrates, have a high ability for membrane transport and play a role in the carbon, iron and nitrogen cycles (Ward et al., 2009). These traits, coupled to the ability for desiccation, motility, biofilm formation and the ability to survive under nutrient limitation (Ward et al., 2009; Hartmann et al., 2015) indicate a potential role in sustaining nutrient cycling in these contaminated soils which potentially could support degradation by specialists. The increase in *Bradyrhizobium* in rapid degradation cluster soils provides a potential mechanism for the increased rate of pesticide degradation as this lineage has been shown to encode phosphodiesterate and phosphotriesterase enzymes and to potentially play an important role in organophosporus degradation (Abd-Alla, 1994). *Burkholderia* has similarly been shown to contain organophosphorus degrading genes (Singh, 2009) and was higher in the rapid degrading cluster. Network analysis indicated that many of the taxonomic groups which were elevated in abundance in the rapid degradation cluster were associated with genes such as nutrient cycling and phosphorus metabolism indicating their key role in the community and potential support for degradation. However, to confirm these arguments, more key OP degrading taxa need to be isolated and their genomic and biogeochemical attributes examined.

### Implications for pesticide efficiency and agricultural use

As well as being supported by the shifts in functional potential and microbial community composition, the differences in potential degradation rates reflected the legacy impact of pesticide usage at these sites. The soils demonstrating increased contemporary rates of *in vitro* CP degradation were soils that historically had developed lower pest control efficiency resulting in a switch to a different, non-OP, pesticide 15 years ago. Surprisingly, samples from site 5H clustered with slower degrading soils from the Mackay site (3) which have not developed high rates of field degradation and on which the OP chlorpyrifos is still applied. Although adjacent samples from the same region at this site showed rapid degradation historically, soils from the site 5H indeed showed slow rates of degradation in laboratory experiments, providing support to the findings of clustering analysis using metagenomic data.

Generally, our analyses suggest a legacy effect whereby the rapid degradation of pesticide was maintained even after its discontinued use 13 years ago, and the development of a microbial community able to adapt to and degrade OP that is still reflected in community function and contemporary OP degradation in the lab. This highlights the long term consequences of pesticide application on soil microbial communities (Singh, 2009; Imfeld and Vuilleumier, 2012) and is contrary to earlier literature suggesting that pesticide application has only a transient effect on community composition (Gevao et al., 2000; Kalam et al., 2004; Imfeld and Vuilleumier, 2012). Such findings are relevant to farmers and the pesticide industry and can aid decisions to select the most efficient pesticide for a given site.

## Conclusion

### A system-wide approach to investigating microbial degradation

Bioremediation studies have traditionally focused on individual organisms and degrading pathways in isolation; however, the use of micro-organisms for bioremediation requires an understanding of all physiological, microbiological, ecological, biochemical and molecular aspects involved in pollutant transformation (Iranzo et al., 2001; Singh and Walker, 2006; de Lorenzo, 2008). Indeed there is a growing understanding that complex microbial communities act as a multispecies metabolic network of “pan enzymes” that collectively allow catabolic breakdown of contaminants within the wider community ecology of the site (de Lorenzo, 2008). By profiling microbial metabolic potential, which includes both genes potentially involved in OP degradation and key soil functions, in pesticide exposed soils we have shown the value of using a system-wide approach and demonstrated that metagenomic profiles can potentially predict the breakdown of chemical compounds. By using metagenome signatures as indicators of degradation potential in exposed habitats and to aid decision support systems for determining the optimum remediation strategy (attenuation, stimulation, augmentation), we provide a conceptual framework for bioremediation that when replicated *in situ* can begin to fully harness emerging ecogenomic tools to improve remediation efficancy.

## Author contributions

TJ conducted experiments, analyzed data and wrote the manuscript, SR conducted experiments and analyzed data, KL, LN, and AI analyzed data, UN conducted experiments, BKS conceived the project and wrote the manuscript.

### Conflict of interest statement

The authors declare that the research was conducted in the absence of any commercial or financial relationships that could be construed as a potential conflict of interest.
