# Supplementary figures and images for: Metagenomic Functional Potential Predicts Degradation Rates of a Model Organophosphorus Xenobiotic in Pesticide Contaminated Soils
Source: Front Microbiol. 2018 Feb 20;9:147. doi: 10.3389/fmicb.2018.00147 (PMC5826299; doi:10.3389/fmicb.2018.00147)

Supplementary Table 3 – Site locations and pesticide history


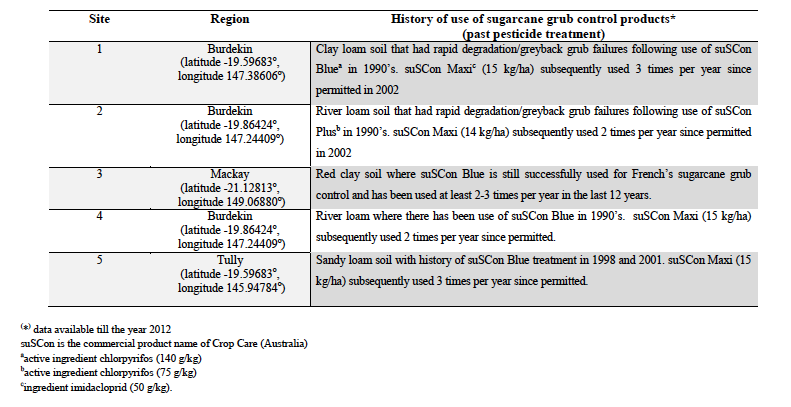

Supplement: Supplementary file 3 [file Table3.DOCX]
