# Supplementary material for: Metagenomic Functional Potential Predicts Degradation Rates of a Model Organophosphorus Xenobiotic in Pesticide Contaminated Soils
Source: Front Microbiol. 2018 Feb 20;9:147. doi: 10.3389/fmicb.2018.00147 (PMC5826299; doi:10.3389/fmicb.2018.00147)
Supplement: Supplementary file 4 [file DataSheet1.DOCX]

A)


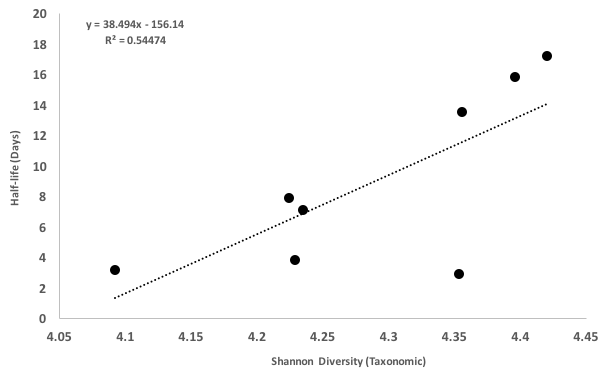


B)


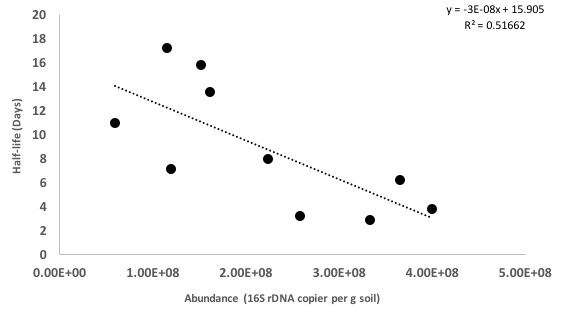


Supplementary Material Figure 1: CP Degradation as a function of A) microbial diversity (Shannon Index) and B) microbial abundance (copies of 16S rDNA per g of soil).


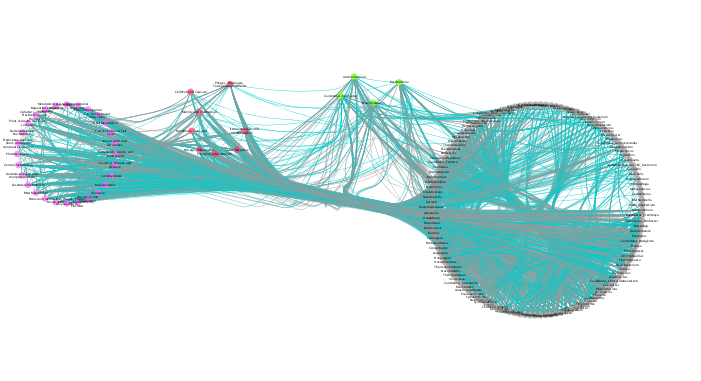


Supplementary Material Figure 2: Network analysis of variable interactions. All edges are statistically significant (p<0.05) based on Maximal Information Coefficient (MIC) score. Pink nodes =metabolic category, dark pink nodes = metabolic categories found to be top drivers of clustering (T-test), grey nodes = taxa (genus), green circles = genera found to be top drivers of clustering (T-test). Black edges = positive interactions, blue = negative interactions.

A)


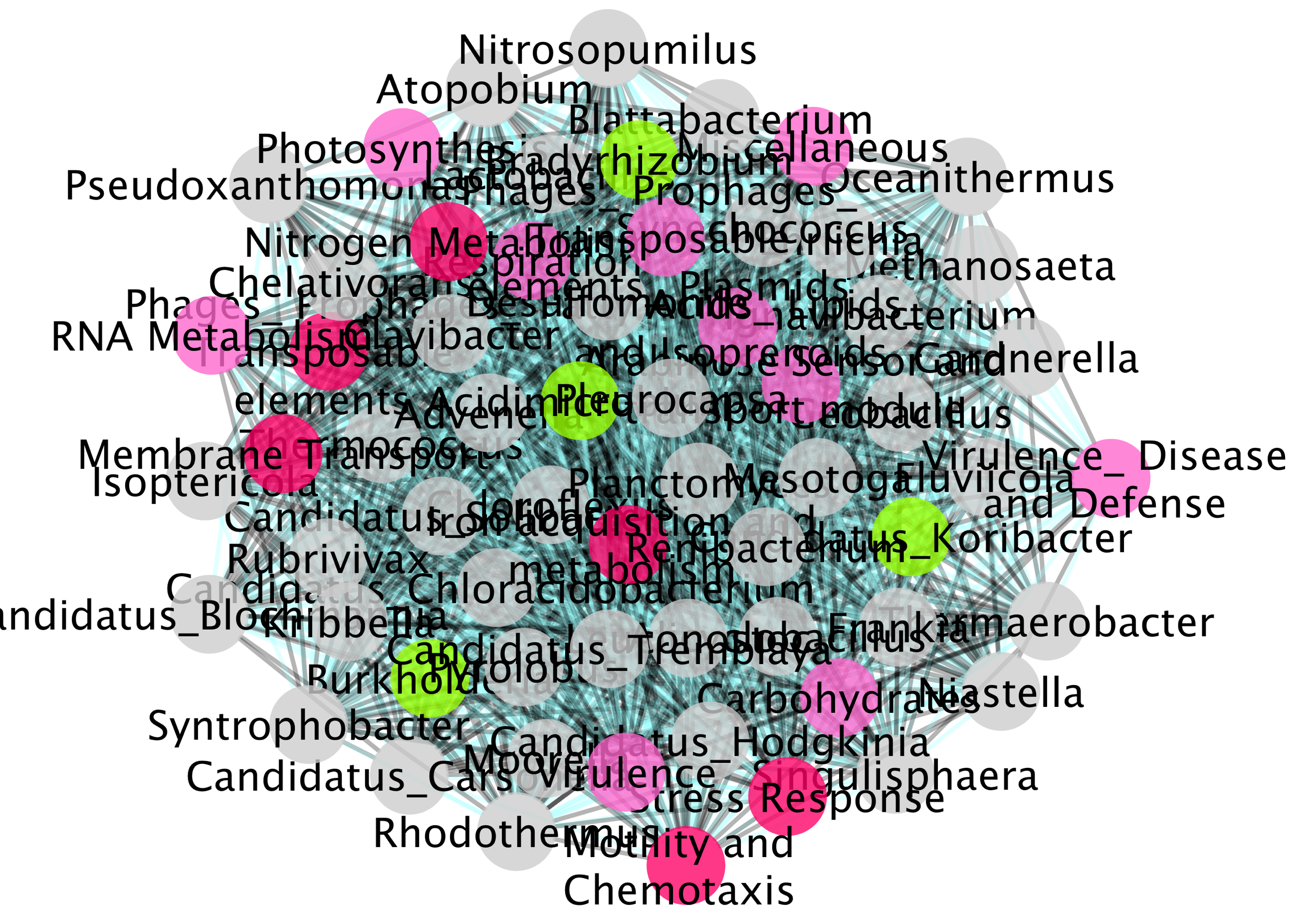


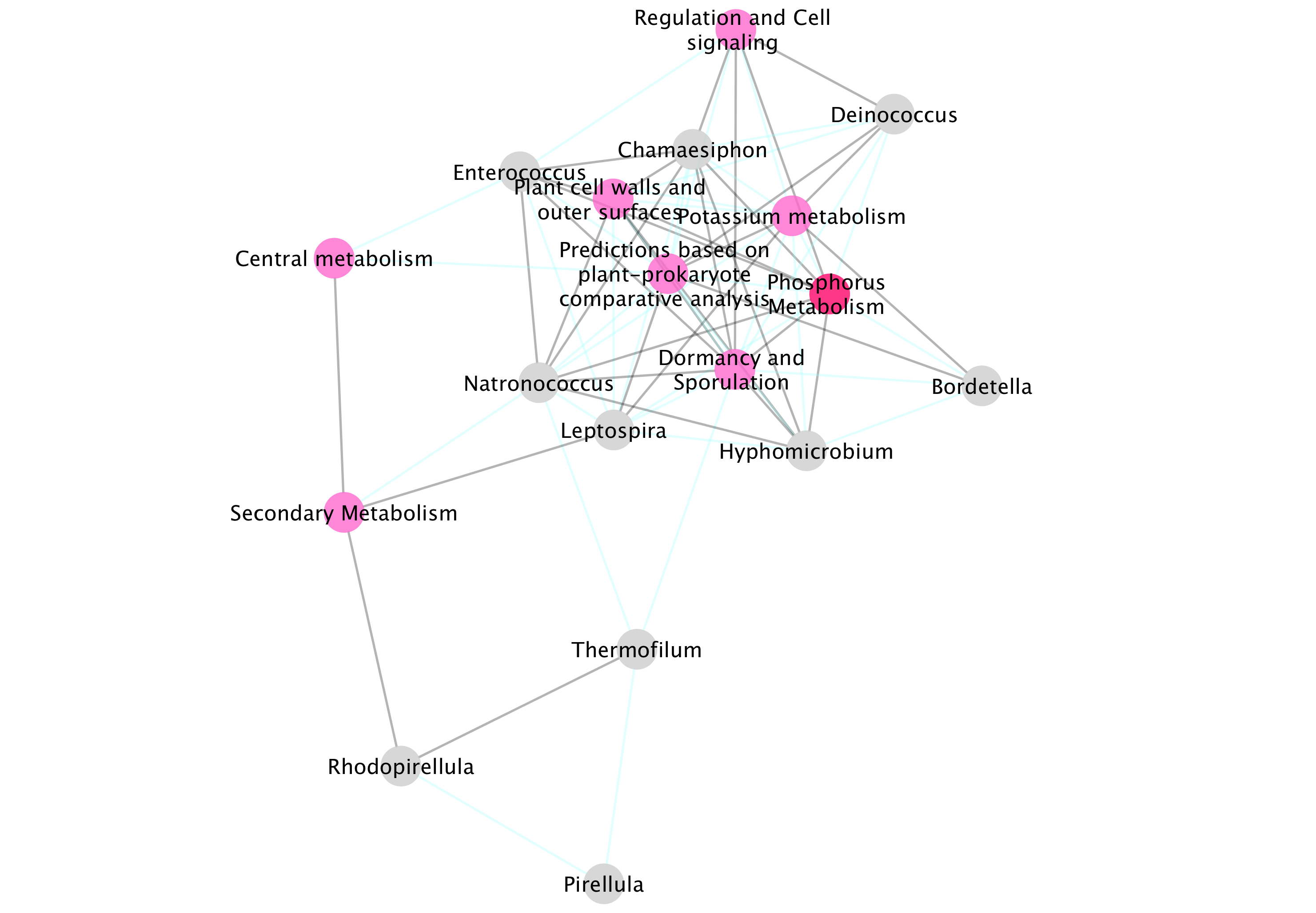


Supplementary Material Figure 3: MCODE analysis of modularity to identify closely associated nodes. The top scoring (A) and second top scoring (B) modules are shown. All edges are statistically significant (p<0.05) based on Maximal Information Coefficient (MIC) score. Pink nodes =metabolic category, dark pink nodes = metabolic categories found to be top drivers of clustering (T-test), grey nodes = taxa (genus), green circles = genera found to be top drivers of clustering (T-test). Black edges = positive interactions, blue = negative interactions.
